# Sticking under Wet Conditions: The Remarkable Attachment Abilities of the Torrent Frog, *Staurois guttatus*

**DOI:** 10.1371/journal.pone.0073810

**Published:** 2013-09-25

**Authors:** Thomas Endlein, W. Jon P. Barnes, Diana S. Samuel, Niall A. Crawford, Ang Bee Biaw, Ulmar Grafe

**Affiliations:** 1 Centre for Cell Engineering, Institute for Molecular, Cell and Systems Biology, University of Glasgow, Glasgow, Scotland, United Kingdom; 2 Kuala Belalong Field Studies Centre, Universiti Brunei Darussalam, Faculty of Science (Biology), Gadong, Brunei Darussalam; University of Sao Paulo, Brazil

## Abstract

Tree frogs climb smooth surfaces utilising capillary forces arising from an air-fluid interface around their toe pads, whereas torrent frogs are able to climb in wet environments near waterfalls where the integrity of the meniscus is at risk. This study compares the adhesive capabilities of a torrent frog to a tree frog, investigating possible adaptations for adhesion under wet conditions. We challenged both frog species to cling to a platform which could be tilted from the horizontal to an upside-down orientation, testing the frogs on different levels of roughness and water flow. On dry, smooth surfaces, both frog species stayed attached to overhanging slopes equally well. In contrast, under both low and high flow rate conditions, the torrent frogs performed significantly better, even adhering under conditions where their toe pads were submerged in water, abolishing the meniscus that underlies capillarity. Using a transparent platform where areas of contact are illuminated, we measured the contact area of frogs during platform rotation under dry conditions. Both frog species not only used the contact area of their pads to adhere, but also large parts of their belly and thigh skin. In the tree frogs, the belly and thighs often detached on steeper slopes, whereas the torrent frogs increased the use of these areas as the slope angle increased. Probing small areas of the different skin parts with a force transducer revealed that forces declined significantly in wet conditions, with only minor differences between the frog species. The superior abilities of the torrent frogs were thus due to the large contact area they used on steep, overhanging surfaces. SEM images revealed slightly elongated cells in the periphery of the toe pads in the torrent frogs, with straightened channels in between them which could facilitate drainage of excess fluid underneath the pad.

## Introduction

Tree frogs stick by wet adhesion in that a fluid film fills the contact zone between the adhesive pad and the substrate [1], [2]. The adhesive mechanism involves both capillarity and hydrodynamic forces [3], though the precise mechanism remains elusive. Their adhesive toe pads have a complex structure, the outer layer consisting of hexagonal epithelial cells separated from each other at their tips [4]. The fluid that fills the adhesive joint is mucus, originating from mucous glands whose ducts secrete their contents into the channels that separate the cells [5]. The mucus is a very watery solution [6] but the exact chemical composition is unknown. The channels serve to spread mucus over the surface of the pad, and can possibly assist in getting rid of excess fluid from underneath the pad. Good adhesion depends upon there being a thin but continuous fluid layer beneath the pad [7]. On the surface of these epithelial cells is an array of nanopillars (300–400 nm in both diameter and height), separated by narrow channels [8]. Previous research suggested that friction forces, also important for climbing frogs, depend on the tips of these nano-pillars making direct contact with the substrate [6].

Much less is known about adhesion and toe pad morphology in rock or torrent frogs. Ohler [9] carried out a preliminary study on the toe pad morphology of torrent-living ranid frogs, demonstrating that the toe pads of *Amolops* had distinct anatomical differences from the typical pattern seen in tree frogs. The only relevant biomechanical work is a preliminary study of adhesion in the Trinidadian stream frog, *Mannophryne trinitatis* [10]. This species is good at adhering to rough wet surfaces, but very poor at sticking to dry rough ones. It also slips on smooth wet surfaces. This suggests that torrent frogs can cope with running water so long as the surfaces are rough.

Brunei, with several species of torrent frog capable of adhering to vertical rocks in waterfalls, and rock frogs capable of fast movement over wet rocks [11], presented a very good opportunity to study these frogs that are clearly specialists in adhesion under wet and flooded conditions. The present study compares the attachment capabilities of a torrent frog species to those of a tree frog species to see first whether there are differences in performance between the two species under different conditions (substrates varying in wetness and roughness), and second what these differences are based upon. For instance, is adhesive performance dominated by the area of contact used by the frogs, or do the two species show a difference in force per unit area that they can generate; i. e. are the toe pads of the torrent frogs more effective under wet conditions?

## Materials and Methods

This was a study of free-ranging animals temporarily brought into a field-based laboratory and later released at their site of capture. Individuals used for electron microscopy where given a lethal dose of Benzocaine. The experimental protocol adhered to the Animal Behaviour Society guidelines for the use of animals in research and was approved by the University of Brunei Darussalam Research Committee (UBD/DVC/32.10) and an export permit JPH/TAD/30 PT 10. We confirm that the species investigated are not endangered or protected.

### Study animals

**Figure 1:**
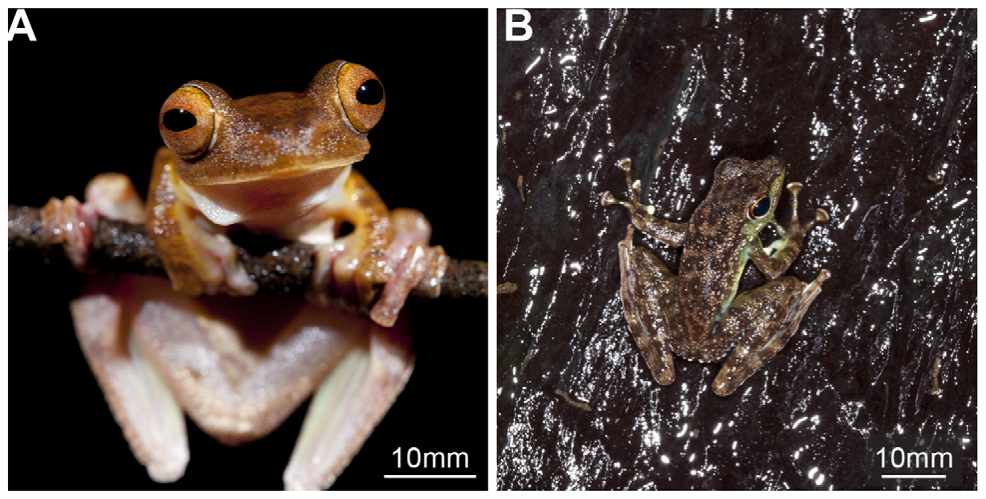
Frog species used in this study. Males of the (A) Harlequin Tree Frog (*R. pardalis*) and (B) the Black-spotted Rock Frog (*S. guttatus*) in their natural habitats.

Our study focused on the comparison of a tree frog and a torrent frog species (Figure 1). Tree frogs are known to be good climbers [2], [12] but, unlike torrent frogs, are not found on rocks where there is fast flowing water. We chose male individuals of the Harlequin Tree frog (*Rhacophorus pardalis*) and male individuals of the Black-spotted Rock frog (*Staurois guttatus*). For an easier distinction between the two, we will refer to *R. pardalis* as the tree frog and to *S. guttatus* as the torrent frog. Both species were abundant around the Kuala Belalong Field Studies Centre, Ulu Temburong National Park (Brunei Darussalam, northern Borneo), where the experiments were carried out during two six-week visits (May/ June 2010 and 2012). The torrent frogs were found near waterfalls on fast flowing streams, where they could be captured on rocks (day) or surrounding vegetation (night). The tree frogs were caught at night on vegetation near small ponds in the forest. Although not identical in either body mass or snout-vent length (SVL), they were the best species match that was obtainable in sufficient numbers in the local area. Body mass was measured using an electronic balance (Mettler), while SVL was measured using callipers. Values are given in Table 1. The frogs were housed in plastic tanks, containing structural elements (rocks, branches and leaves) and ca. 1 depth of water. After the experiments, the frogs were released at the sites where they were captured.

**Table 1:** Masses and lengths of the two species used in the experiments.

Species	Mass (g)	SVL (mm)	*N*
*R. pardalis*	4.0±0.4	45±2	34
*S. guttatus*	2.7±0.3	34±1	39

SVL, snout-vent length.

### Measurement of the attachment abilities of the frogs

**Figure 2:**
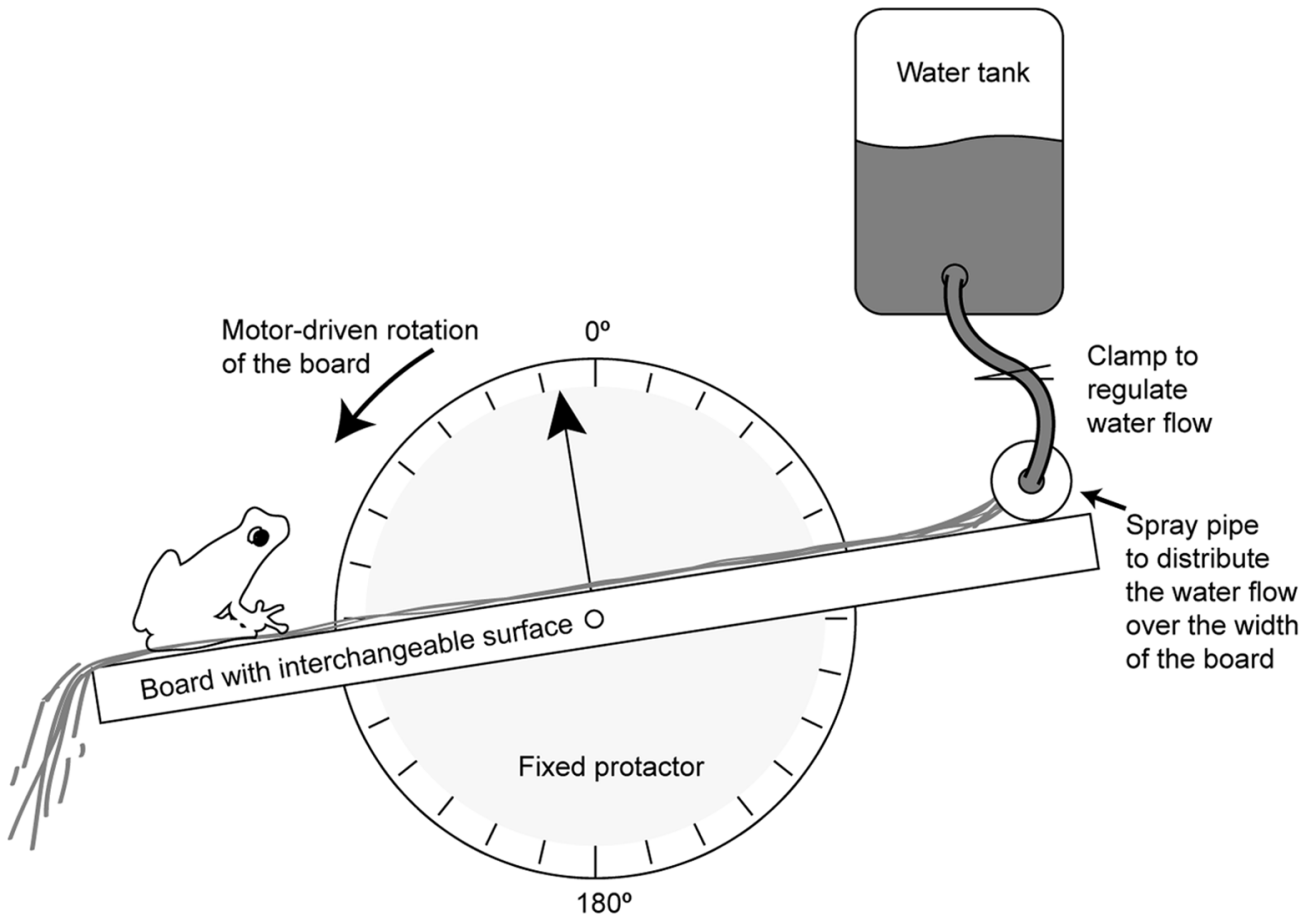
Diagram of the key elements of the tilting platform. Not drawn to scale.

Attachment ability was measured on a rotating platform, using a technique previously used by [1], [7] and [13]. Frogs were challenged to cling onto the platform which could be rotated from an initial horizontal orientation, through the vertical to an upside-down orientation (Fig. 2). A custom-built geared motor system, adapted from a Stuart SB3 rotator (Bibby Scientific Ltd, Stone, Staffs, UK), ensured a constant rotation speed of 4
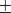
1° s^−1^. The platform consisted of a wooden base (20
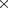
30) with the possibility of mounting different surfaces on top of it. The surfaces used in our experiments were a smooth Perspex sheet, a fine rough surface (30 μm aluminium oxide polishing discs, Ultratec, USA) and a coarse rough surface (custom-built by gluing a single layer of Ballotini glass beads (1125
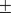
125 μm) onto a Perspex sheet). As the surfaces were made from different materials, the surface chemistry between the surfaces may have differed. Previous work (Barnes, unpublished) has shown that tree frogs stick equally well to glass, Perspex and hydrophobic leaves, so it is reasonable to assume that the effects observed here are primarily due to changes in wetness and roughness.

We also equipped the platform with a running water system to flood the surfaces in a controlled manner (Fig. 2). The water flow system was gravity-fed by a large storage tank, placed ca. 1.2 m above the platform. Two hoses led from the tank to a short pipe placed across the top end of the platform where the water poured out from small holes drilled into the pipe. The flow rate was controlled by adjustable clamps on both hoses, and the flow was measured prior to and following each experimental run. We used three flow regimes: zero flow (i. e. ‘dry’), a low flow rate (ca. 50 mL min^−1^, i. e. ‘wet’) which wets the platform, and a fast flow rate (ca. 4000 mL min^−1^, i. e. ‘flooded’), both within the range of flow rates found in the torrent frog's natural habitat. The water formed a more or less uniform layer across the surfaces, and stayed attached to it until the platform was close to the upside down orientation (ca. 170°).

Frogs were placed onto the platform, starting from a horizontal position (0°). In the majority of cases, frogs that initially faced downhill would turn so that they faced uphill as platform rotation progressed. We recorded the angle at which the frogs showed an initial slip and when they were completely dislodged from the platform. From those angles we calculated the maximum friction and adhesion forces using trigonometry. In cases where the frog slipped completely off the platform below 90°, the values for the fall angles were set equal to the slip values. In cases where the frogs showed an initial slip beyond 90°, the slip angles were set to 90°, since this would be the orientation where maximum friction would occur. The angles were measured by reading the position of a needle mounted on the platform against a 360° protractor (estimated measurement error was below 2°).

Each individual frog was tested ten times on all three surfaces and all three flow regimes. The order of each combination of surface roughness and flow regime was randomised to avoid order effects. The frogs were given time to recover between trials. In a few cases, where the performance of the frog deteriorated significantly, we stopped the experiment and replaced the frog with a fresh individual.

### Measurement of contact area

In order to see which body parts of the frogs were in contact with the substrate, we used a special illumination technique on a transparent glass surface (dimensions: 200
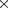
300
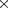
4 mm). The illumination technique was adapted from Betts [14] and consisted of arrays of LEDs (Tru Opto, ultra-bright 5 mm, narrow angle) attached to each of the four edges of the glass plate so that the light entered the glass plate and was totally internally reflected within it (refractive index 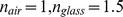). Normally, very little light escaped from the sheet. However, as soon as an object with a similar or higher refractive index to the glass (like the watery frogs' mucus, 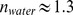, or the toe pad tissue 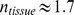) came into contact with the glass, light escaped at this spot and resulted in a bright spot. Like the platform described above, the glass platform was rotated (in this case manually) from a horizontal to an upside down orientation at a velocity of 3.8
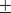
1.1° s^−1^. A digital video camera (Basler A602f, Ahrensburg, Germany) was mounted onto the platform and rotated with it in order to film the ventral body area of the frogs in contact with the glass surface and the protractor that monitored the angle of tilt of the platform. The video recordings (25 frames^−1^) were captured using Streampix (Version 3, Norpix Corp.) and analysed frame by frame using custom-built Matlab routines [15]. The routines converted the frames into binary images based on a threshold value. The resulting images comprised white areas (where the contact occurred) against a black background.

### Force measurements on individual toe pads, belly and thigh skin

For both species of frog, adhesive and friction forces for various adhesive body parts were measured using a custom-built force transducer. These body parts comprised belly and thigh skin (used by the frogs to aid their attachment on steep and overhanging surfaces) in addition to the adhesive toe pads. They were tested under both dry and wet conditions on surfaces of different roughness (smooth, 0.3 μm and 16 μm average particle size of polishing discs; Ultratec, USA). Pads were tested under ‘wet’ conditions by pipetting 10 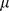
L water onto the body part prior to the measurements, while surface roughness was varied using cut out pieces of the polishing discs.

The experimental set-up was similar to that used in previous single toe pad studies on tree frogs [16]. Held in position within a plastic Petri dish using foam cushioning, frogs were positioned upside-down underneath a two-dimensional force transducer by means of a micromanipulator to allow specific body parts to come into contact with it. For toe pad measurements, the actual force plate consisted of a 1 thick piece of flat polyethylene (15
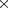
15 mm) that provided a smooth surface for toe pad contact (self-adhesive pieces of polishing disc as described above were attached to the force plate for the measurements on rough surfaces). For belly or thigh skin measurements, the force plate was a circular, curved glass surface (18 mm diameter, radius of curvature 9 mm), a curved surface being more suited to measurements on extended skin areas. The area of contact during such force measurements was measured using the same technique as for whole frogs described above, an array of LEDs surrounding the force plate in such a way that totally internally reflected light only escaped where contact was made, the bright spot representing the contact area being filmed with a digital video camera (Basler A602f, 10–30 frames^−1^).

Forces were measured in two dimensions, lateral (which measured friction) and normal (which measured adhesion). The applied vertical or horizontal movements were made manually by turning the knob of a micromanipulator and were recorded by means of a potentiometer. These inevitably led to some variation between different movements, means and standard deviations being as follows: amplitude 2.9
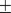
0.7 mm, velocity 1.4
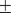
1.1° s^−1^. A custom-built LabVIEW interface (National Instruments, Austin, Texas) was used to display and save force recordings, which were retrieved from the transducer via a portable data acquisition device (NI 9237, National Instruments, Austin, Texas). The video recordings and the force data acquisition were synchronised using a stop trigger pulse from a manual switch.

### Scanning Electron Microscopy

For a closer inspection of the adhesive toe pads and other areas of ventral skin used by the frogs to aid their adhesion on steep and overhanging surfaces (belly and thigh skin), a few individual frogs were sacrificed using a lethal dose of Benzocaine. Fore- and hind limbs were severed at the base of the wrist or ankle, respectively, while belly and thigh skin samples were obtained by carefully cutting out rectangular pieces of skin using a scalpel. All specimens were fixed in a solution of 2.5% gluteraldehyde buffered at pH 7.4 for 24 h. After washing in distilled water, specimens were dehydrated in an alcohol series and critical point dried. Samples were mounted and gold-coated before viewing with a JSM-7500F scanning electron microscope (JEOL UK Ltd.).

### Statistics

All statistical tests were carried out using the statistical toolbox in Matlab (v2012a, Mathworks Corp., USA). Data for contact area was extracted at angles of 45° intervals from 0° to 180°, which allowed us to draw statistical comparisons between these categories.

For comparisons between two samples we used the Mann-Whitney U-test. For each test we provided the sample size (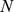), the sum of the ranks (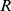), the computed z-statistics (only for large sample sizes) and the error probability (*p*). Test results are either highlighted in the text or summarised in tables listed in Supplementary Materials S1. For the plots, we have indicated a significant difference between two samples using the ‘*’-symbol if 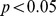, ‘**’ if 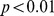 and ‘***’if 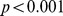. If a test just failed to reach the significance level, we stated the computed p-value, e. g. 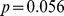. Multiple tests on the same set of data were adjusted using the Bonferroni correction.

Where we have plotted data using box plots, the median value is represented by a line, and the 25^th^ and 75^th^ percentiles by the box. The plotted ‘whiskers’extend to the adjacent values, which are the most extreme data values that are not outliers. Points are drawn as outliers if they are larger than 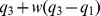 or smaller than 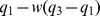, where 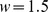 and 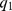 and 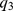 are the 25^th^ and 75^th^ percentiles, respectively. Where we have averaged data, the mean is given together with the standard deviation, if not stated otherwise.

## Results

### Effect of surface roughness and wetness

**Figure 3:**
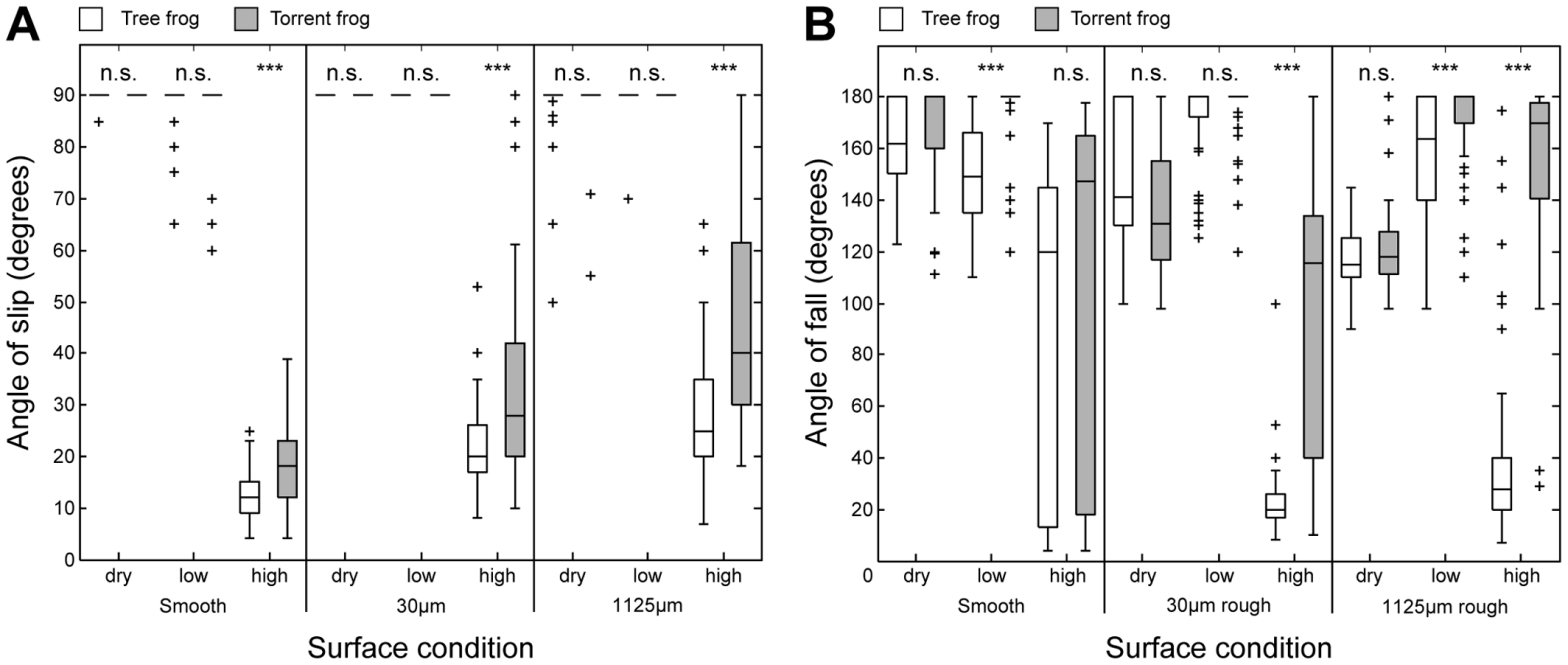
Attachment performance of the two frog species under varying conditions. Comparison of (A) slip angles and (B) fall angles between the tree frog (*Rhacophorus pardalis*) and the torrent frog (*Staurois guttatus*) on different wet (‘low’& ‘high’flow rate) and rough surfaces. The details of the statistical tests between the two frog species are listed in Tables S4 and S6 in Supplementary Materials. Intraspecific differences of frog performance on both different surfaces and under different flow regimes are listed in Tables S1 to S3 and S5 in Supplementary Materials S1.

Frogs were challenged to cling onto smooth, fine rough (30 μm diameter particles) and coarse rough (1125 μm diameter particles) surfaces attached to a rotating platform. Each surface was used under three flow regimes: ‘dry’ ‘low flow rate’and ‘high flow rate’ These data allowed us to examine both the effects of changing flow rate on each surface and *vice versa*. Figure 3 shows the slip and fall angles of the two frog species on the platform and a comparison between them. Results of the statistical tests are listed in Tables S1 to S6 in Supplementary Materials S1.

We will begin by examining the performance of the tree frog (Figure 3, white boxes), which forms the reference for judging the performance of the torrent frog. Slip angles are a measure of the frog's friction force, though are limited to the mass of the frog, since maximum values are given by an absence of slipping at 90°. On the dry and wetted surfaces (low flow rates), very few of the tree frogs (*R. pardalis*) slipped at angles below 90°, regardless of surface roughness. Only on the flooded surfaces (high flow rates) did performance decrease dramatically (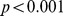, Test No. 3 in Table S1 in Supplementary Materials S1). Lowest slip angles occurred on the smooth surface, but recovered slightly with increased surface roughness (Figure 3A).

Fall angles, on the other hand, are a measure of the frog's adhesive force, the force normal to the surface. They too are limited to the mass of the frog, since maximum values are given by the frog adhering in an upside-down position (180°). Although, as Figure 3B shows, they broadly reflected the pattern of the slip angles, there were differences. For instance, the fall angles achieved by the tree frogs on a smooth dry surface (median value 161°) decreased both with increasing roughness and with increased rate of flow (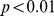, Tests Nos. 2, 3, 10 and 11 in Table S2 in Supplementary Materials S1). For the two higher roughnesses, however, wetting the surface increased adhesion (i. e. resulted in a higher fall angle) (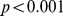, Tests Nos. 4 & 7 in Table S2 in Supplementary Materials S1). Finally, under the flooded condition, performance dropped to low levels on both of the rough surfaces (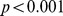, Tests Nos. 6 & 9 in Table S2 in Supplementary Materials S1).

Identical procedures to those just described for the tree frog were carried out on the torrent frog, *S. guttatus* (Figure 3, grey boxes). On all three surfaces under both dry and low flow rate conditions, the frictional performance of the torrent frogs was excellent, very few individuals slipping before 90°. Indeed, as far as slip angles are concerned, the performance of the two species could not be separated statistically on any surface under either dry or low flow rate conditions (Figure 3A). At a high flow rate, however, the performance of the torrent frogs was much worse, particularly on a smooth surface, but, with increasing surface roughness, it improved and went back to maximum in some cases on the coarse rough surface (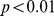, Tests Nos. 16–19 in Table S3 in Supplementary Materials S1). When these data are compared to the equivalent data for tree frogs, it can be seen that the torrent frogs perform better on all three surfaces under the fast flow rate condition (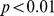, Tests Nos. 3, 6 and 9 in Table S4 in Supplementary Materials S1; Figure 3).

Fall angles for *S. guttatus*, which were high on a smooth dry surface, significantly decreased when surface roughness was increased (Tests Nos. 10–12 in Table S5 in Supplementary Materials S1). However, when there was some water present (low flow rate), performance recovered, median values on all three surfaces being 180°. For the high flow rate conditions, performance was variable on the smooth and 30 μm surfaces, but stabilised at a median of 170° on the roughest surface. Indeed, it is at this high flow rate on the rough surfaces that the torrent frogs excelled in comparison to the tree frogs, the difference between the two species being most dramatic on the roughest surface (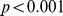, Tests Nos. 6 & 9 in Table S6 in Supplementary Materials S1). The remarkable ability of the torrent frog, *S. guttatus*, to remain attached to these rough surfaces when water is pouring over their bodies can best be appreciated by watching Video S1.

To summarise, there was no significant difference in performance between the species on all the dry surfaces. However, differences appeared on some of the wet surfaces (low flow rate) and were most dramatic under the high flow rates on the two rougher surfaces. The results clearly indicate that the torrent frog (*S. guttatus*), in contrast to the tree frog (*R. pardalis*), is extremely well adapted to adhering to rough surfaces under flooded conditions.

### Contact area on a dry, smooth surface

**Figure 4:**
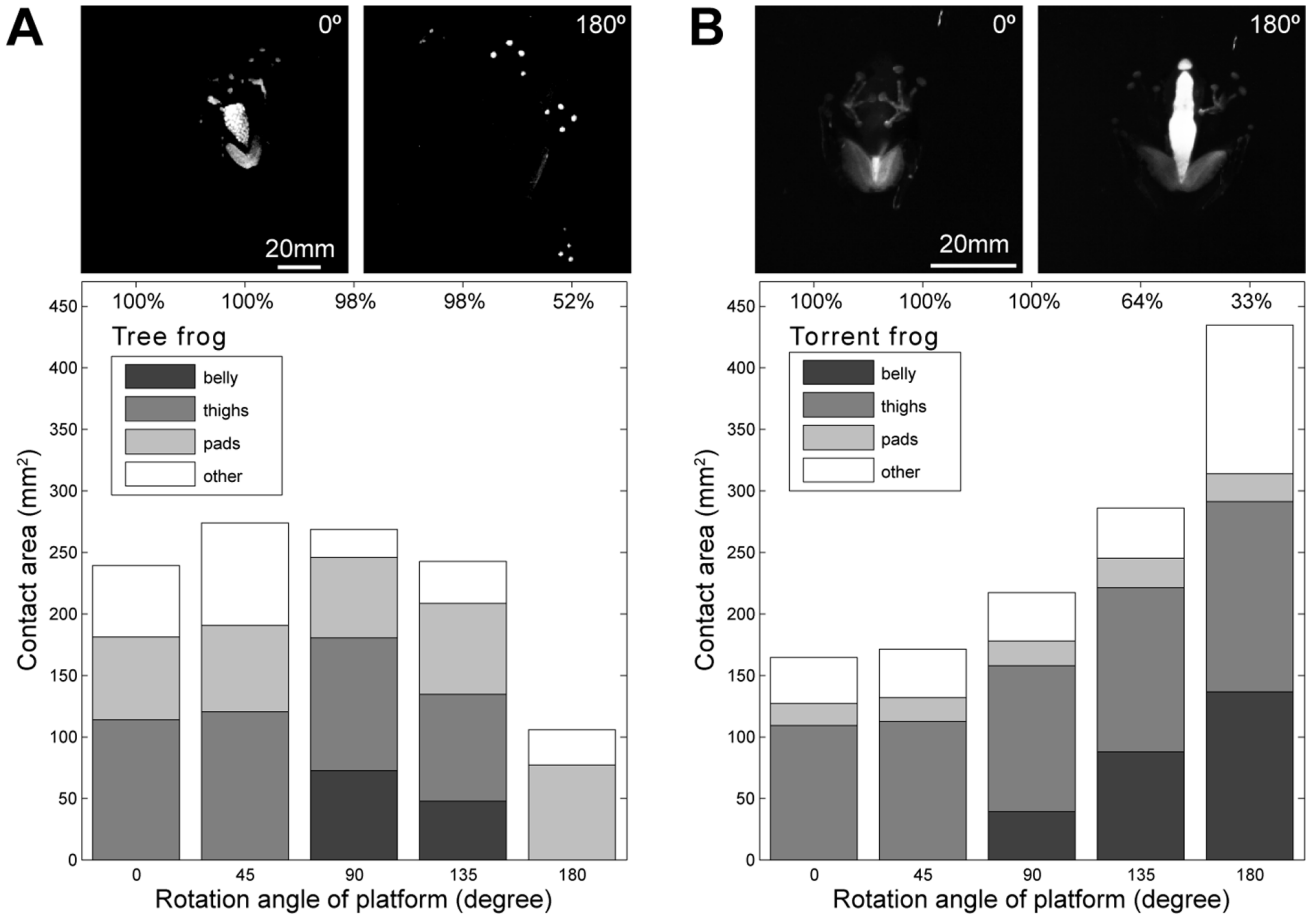
Contact areas of the two frog species at 5 tilting positions. Using a special illumination technique (see Materials and Methods), the contact area of ventral body parts (toe pads, belly, thighs and uncategorised areas) of (A) the tree frog (*R. pardalis*) and (B) the torrent frog (*S. guttatus*) was measured at 0°, 45°, 90°, 135° and 180°. The photos at the top are images of the frogs at horizontal (0°) and inverted (180°) tilting positions. The plots represent medians of 42 trials from 6 frogs (tree frogs) and 33 trials from 6 frogs (torrent frogs), the percentages at the top representing the proportion of frogs still attached at each tilt angle.).

In order to quantify contact area and to see which parts of the frog's body would come into contact over the course of the rotation of the platform, we filmed the frogs from the ventral side through a transparent (dry and smooth) illuminated glass surface so that areas of contact were highlighted (see upper images of frog contact areas in Figure 4). Such videos also provided information on the behavioural strategies used by the frogs as they attempted to hang on to the platform at increasing angles of tilt.

In the tree frog, *R. pardalis*, there was little change until the angle of the platform reached ca. 70°. From this point on, legs were spread out sideways, the spread also having a fore-aft component. This was forwards in the case of the fore limbs and backwards in the case of the hind limbs. Typically, as in Video S2 or Figure S1, this occurred in two ‘steps’ an initial small spread at around 70° followed by a second larger spread at around 135°, similar to the behaviour observed in a different tree frog species [17]. Additionally, if the frog were facing downhill initially, the frog would rotate to face uphill at an angle between 90° and 135°. Finally, in 48% of the trials (20/42), the frog fell from the platform between 135° and the fully inverted position. In terms of contact area, thigh and, to a lesser extent, belly skin (plus other areas of ventral skin) were used as well as the toe pads at low angles of tilt (Figure 4A). Then, at an angle in excess of 135° (or at time of body reorientation for frogs that were initially facing downhill), body contact would often be lost so that the frog was hanging on by its toe pads alone (Figure 4A, top right image). In such cases, the toe pads on both, the fore- and hind limbs would continuously slide proximally and would be replaced in an extended position at intervals. In addition, the pads of the fore limbs would also slide towards the centre of each hand. Such behaviour gives the observer the impression that the frogs are ‘dancing’on the inverted platform (see Video S2). Only in 24% of trials (10/42) were the tree frogs able to maintain body (as opposed to toe pad) contact using their thigh and belly skin up to and including the upside-down orientation.

The torrent frogs (*S. guttatus*) used a similar amount of total ventral body area when they were resting against a horizontal surface (0 rotation of the platform). Pad contact area was, however, much smaller (approximately 25% of that in the larger tree frogs). As in the tree frogs, there was little change in behaviour until the platform rotation passed 70°, at which point total contact area began to increase, mainly through use of belly skin. At angles between 90° and 135°, the limbs were spread out sideways as in the tree frogs (though to a lesser degree) and frogs initially facing downhill would reorientate to face head-up (Video S3 or Figure S1). We tested whether this turning behaviour influenced the amount of contact area before and after the frog's re-orientation. In contrast to tree frogs, nearly all individual torrent frogs managed to reattach their belly skin after they turned around and increased their contact area even further (Mann-Whitney U-test: 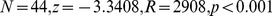). This ability might be crucial when climbing areas of fast flowing water, where quick re-attachment is a vital necessity.

To summarise, *S. guttatus* increased contact area with the substrate as they were rotated from 0° to 180°, mainly by the use of the belly skin, while the tree frogs exhibited a decline in contact area, so that the majority were hanging on by their toe pads alone (Figure 4A, B). Note, however, that this increasing use of the body did not give torrent frogs any advantage on this smooth dry surface, as only 33% (11/33) maintained their attachment until 180°, compared to 52% of the tree frogs. Total pad area was also clearly smaller in the torrent frogs, even when the difference in body size is taken into account, but they did show a greater ability to recover body contact area after a behavioural manoeuvre. Finally, limb spreading was a common behavioural feature exhibited by both frog species when rotated. Thus, it illustrates an important strategy for enhancing attachment on overhanging surfaces [17].

### Force measurements on individual pads, belly and thigh skin

As described above, torrent frogs use parts of their ventral body surface in order to stay attached to overhanging surfaces. In contrast, tree frogs often relied solely on their toe pads. It was therefore desirable to measure the adhesive and friction forces of the body parts used by the frogs to stay attached. We used a 2D-force-transducer with a transparent surface attached to the end as a probe. This allowed us to record contact area using the LED system described above, while simultaneously measuring adhesion and friction forces. We tested the different body parts under two conditions: natural condition (referred to here as ‘dry’, and with added water (referred to here as ‘wet’.

**Figure 5:**
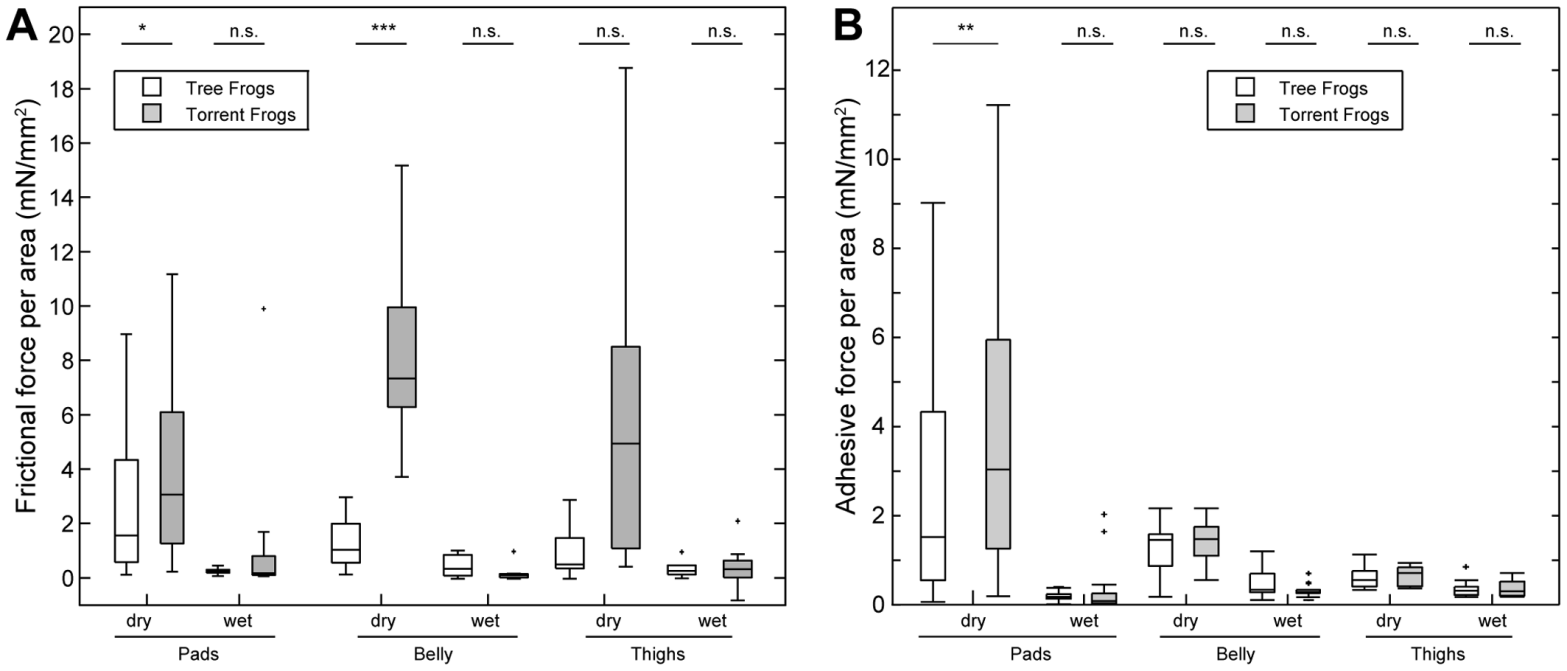
Friction and Adhesion forces per area of different body parts. Statistical differences are denoted as follows: ‘*’ 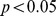, ‘**’: 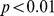, ‘n.s.’: not significant.

Figure 5 shows a comparison of the shear and adhesive stress of the different body parts under varying conditions. Under dry conditions, the toe pad forces of both species greatly exceeded the forces per unit area previously recorded in other frogs. For instance, median adhesive forces of 1.5 mN mm^−2^ for the tree frogs and 3.0 mN mm^−2^ for the torrent frogs can be compared to 0.7–1.0 mN mm^−2^ for the adhesive forces of several species of hylid tree frogs [7]. Torrent frogs adhered significantly better than the tree frogs on the dry surface of the force plate in respect of both adhesion (normal forces) and friction (shear forces) (see Test No. 1 in Table S7 in Supplementary Materials S1 and 10, respectively). Under wet conditions, the force levels of the pads in both species dropped to a tenth of their adhesive capability under dry conditions (see Test No. 2 in Tables S7 and S8 in Supplementary Materials S1, respectively). This was not entirely unexpected, since similar results have been obtained for the tree frog *Litoria caerulea* by [18]), and are thought to reflect a lowering of the capillary forces resulting from an increase in the thickness of the fluid layer under the pad.

Under dry conditions, both belly and thigh skin made a significant contribution to adhesion and friction in both species. Friction forces from these body areas were particularly high in the torrent frogs and appeared to be due in part to the wrinkling of the skin against the force plate, the skin being rather loosely attached to underlying tissues at these points. As with the toe pads, forces were much lower under wet conditions for both body areas in both species (see Tests Nos. 3–6 of Table S7 in Supplementary Materials S1).

In summary, the toe pads of both species produced high adhesive and frictional forces in comparison to other frogs previously studied, with significant contributions also coming from ventral parts of the body skin (thighs and belly). However, under wet conditions, values were significantly reduced. Additionally, preliminary data using rough surfaces (not shown) also produced low levels of adhesion, particularly under wet conditions.

### Scanning Electron Microscopy

**Figure 6:**
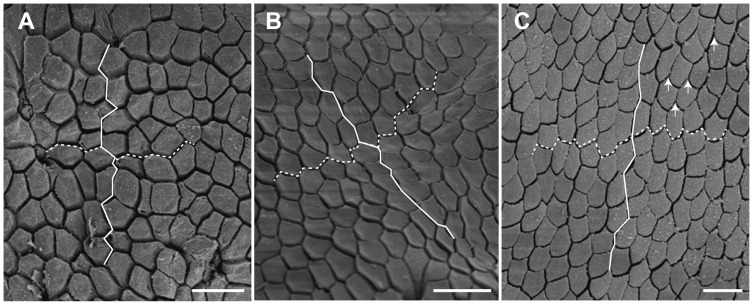
Scanning electron micrographs of toe pad epithelia in different frog species. (A) the tree frog *R. pardalis*, (B) the torrent frog *S. guttatus* near the edge of pad, and (C) the torrent frog, *Odorrana hosii*. White solid lines illustrate shortest routes to the edge of pad. White dashed lines are routes across the pad. Arrows show examples of the pointed ends of the epithelial cells of *O. hosii*. Scale bars: 20 μm.

**Figure 7:**
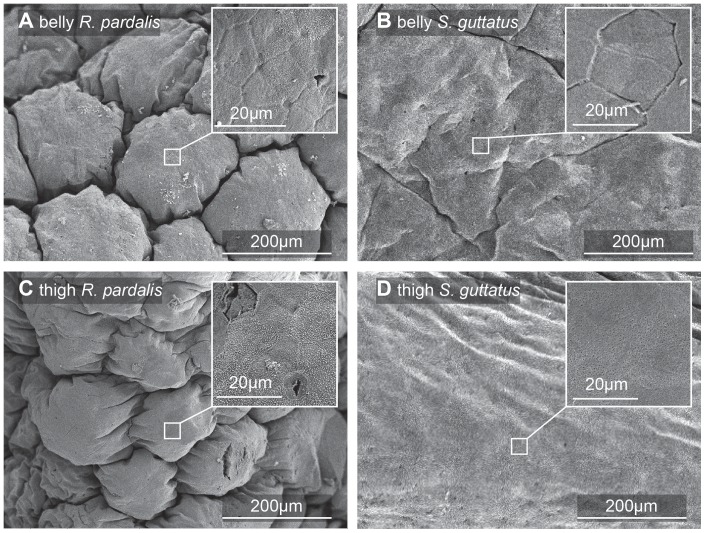
Scanning electron micrographs of ventral body skin. Belly (A,B) and ventral thigh epithelium (C,D) of the tree frog (*R. pardalis*, left column) and the torrent frog (*S. guttatus*, right column). Insets show structures at higher magnification.

In the present study, we found that unrestrained torrent frogs adhered better than tree frogs to rough surfaces under wet conditions. Are there any morphological adaptations which could help to explain their better performance? Most tree frogs have hexagonal cells that are uniformly shaped (width-to-length ratio of the hexagons close to 1). However, previous work on torrent frogs [9] showed that the toe pad epithelium of ranid torrent frogs from a number of genera consisted of elongated cells (i. e. cells that deviate from a regular hexagonal shape), resulting in the channels between them providing shorter and straighter pathways from the centre of the toe pad to its edge. This has been presumed to be an adaptation for better drainage of water from under the toe pads in their flooded environment. Here we show images of the toe pads of the tree frog and torrent frog under study (Figure 6A, B), together with an image of another torrent frog species, *Odorrana hosii*, also found in the Brunei rainforest in the region of fast flowing rivers (Figure 6C). All three images are at the same magnification and are oriented so that the nearest edge of the pad is approximately at the top of the page. *O. hosii* shows the elongated shape typical of torrent frogs (width-to-length ratio smaller than 1 in the radial direction). Although usually surrounded by six other cells, the cells are not hexagons, as they mainly have curved rather than straight edges. Their ends are often pointed, especially the ends nearest the edges of the pad (see arrows in Figure 6C). As you can see from the line drawn on the image, the channels directing water towards the edge of the pad are almost straight. In our tree frog species, the pattern of epithelial cells is a lot more variable than the patterns of regular hexagons illustrated in [13] for hylid tree frogs. Most of the cells do, however, have six neighbours and straight edges, and so are irregular hexagons. Channel lengths in the direction of the edge of the pad are thus not shorter than across the pad (compare lengths of white solid and dashed lines). In our torrent frog species (*S. guttatus*), cell shape seems to vary with the region of the pad. In central regions, there is a tendency towards a pattern of roughly regular hexagons, but peripherally they are more elongated, the overall pattern being intermediate between those of the tree frog and *O. hosii* (Figure 6B). In such regions, channel lengths in the direction of the pad edge are relatively short, certainly shorter than across the pad (see lines on Figure 6B). We have also examined the structures of thigh and belly skin in both our species (Figure 7). Like the toe pad epithelium, the epithelium of the ventral surface of the tree frog (belly and ventral thigh skin) was subdivided by deep channels at intervals of about 200 μm, giving a quilted appearance, the cells being irregularly hexagonal, ca. 20 μm in diameter. In contrast, the belly and thigh skin of the torrent frog was relatively smooth, again consisting of approximately hexagonal cells of ca. 20 μm diameter. It is however unclear how these structures aid adhesion or friction.

## Discussion

The first part of this paper describes behavioural experiments which show that, while both representative tree (*R. pardalis*) and torrent (*S. guttatus*) frogs adhere equally well to smooth, dry surfaces, the torrent frog species excels on rough surfaces covered by fast-flowing water, i. e. in conditions that replicate their natural habitat of wet rock in the immediate vicinity of waterfalls [19]. The second part of this paper seeks an explanation for the torrent frog's remarkable ability by visualizing contact area at different angles of tilt for both species, and by measuring adhesive and shear forces from single toe pads and portions of ventral skin used by the frogs to adhere to steep and overhanging surfaces. Additionally, we used scanning electron microscopy to look for possible structural adaptations for adhesion and friction.

### Effect of surface wetness and roughness

Both frog species were affected in their attachment abilities by surface roughness and the amount of water on the surfaces. When no water was involved, frogs performed best on the smooth surface. We speculate that, under these conditions, the contact area can be maximised by the secreted mucus of the frogs alone. The attachment ability of both frog species decreased on dry surfaces when roughness increased. We believe that this could be due to insufficient mucus production to fill all the asperities of a rough surface, so that real contact area and therefore adhesive forces decreased. This is supported by the fact that additional water (under the low flow rate regime) helped to increase performance significantly. However, at the highest flow rates, friction forces dramatically decreased, presumably because of the lubricating effect of the water flow, with follow-on effects on adhesion. It was here, on the 30 μm and 1125 μm surfaces, that the torrent frog outperformed the tree frog. Indeed, the difference on the roughest surface was dramatic. Almost all of the tree frogs were swept off the platform before the angle reached 90°, while the torrent frogs stayed attached until the platform was almost upside-down. Thus, on these rough, wet surfaces, torrent frogs clearly have better attachment (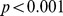, Test No. 9 in Table S6 in Supplementary Materials S1). The few tree frogs that remained attached on such surfaces beyond 90° (7 out of 50) did not perform quite as well as the torrent frogs (median fall angle of 145° for the tree frogs compared to 172° for the torrent frogs), but this difference is not statistically significant (Mann Whitney U-test, 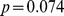) probably because of the small sample size. So, whether the torrent frogs have better adhesion as well as having better friction on these rough, wet surfaces remains unclear.

### Adhesive strategies

As our results indicate, the two species exhibit rather different behaviours when adhering to steep and overhanging smooth, dry surfaces. Although both species use part of their ventral body surface to adhere at low angles of tilt, this is no longer true at angles between 135° and 180°. In the tree frog, the body tends to detach, so that, in the majority of trials, the frogs are hanging on by their pads alone. The limbs are spread out sideways, but slide towards the body before being replaced by rapid limb extensions. The frogs appear to ‘ance’on the inverted glass surface (see Video S2). As analysed in another frog species, this limb spreading behaviour seems to be a mechanism for both increasing the available adhesive force and reducing the chance of unintended toe pad detachment, since spreading the pads keeps the pad/surface angle low. At low pad/surface angles, adhesion is proportional to the area of the surface, while at high angles it is proportional to the length of the peel zone [20] in accordance with peeling theory [17]. Additionally, toe pad detachment occurs readily at high pad/surface angles [3]. In contrast, the torrent frogs, although they also spread their limbs (but to a lesser extent than the tree frogs), tended to increase the contribution of thigh and belly skin at high angles of tilt. These different strategies did not result in significantly different adhesion capabilities on these smooth, dry surfaces, but could be significant on wet, rough ones. This remains an area of uncertainty, since our experimental method for measuring contact area could not be applied to such surfaces.

### Correlating force and contact area

The data on contact area and force per unit area can be combined and compared to the adhesive and friction forces needed to support the frogs at different angles of tilt. Table 2 shows such an analysis. For friction forces, we measured contact area for each species at 9°; for adhesion forces we measured it at 180°. We have used median values rather than means, so that the results are not affected by the occasional rogue value (our force measurements occasionally produced very high values, which we had no reason to exclude). Total force (area 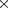 force per unit area) for each surface (toe pads, ventral thigh skin and belly skin) were summed to give the maximum available adhesive and friction force available under both dry and wet conditions. In all but one case, the total force was more than enough to support the frog. This provides a degree of validation for our force measurements. The one exception (tree frog adhesion under wet conditions) is entirely due to the use of median values rather than means, as the use of mean values gave a total force of 88.0 mN, more than enough to support the frog. These results suggest that the frogs have significant safety factors, in some cases far higher than would have been expected. Interestingly, even higher values can be calculated for gecko adhesion by multiplying the maximum adhesive force of a single seta with the number of such setae on a gecko's subdigital pads [21], [22]. In both cases, such values are probably overestimates, since the frogs often struggle to adhere at angles near 180°, and there is no evidence that a tokay gecko can actually support the weight of two humans as the calculations suggest! Of particular relevance to this study is first that the safety factors of torrent frogs are higher than those of the tree frogs for the equivalent experimental situation and second that the toe pads have a relatively minor role compared to ventral body skin in the adhesion/friction of torrent frogs. On wet rock, the torrent frogs progress by a series of jumps, so body skin can play a role in attachment in a way that would be impossible in a walking animal.

**Table 2:** Combining the contact area with the force per area measurements to calculate maximum attainable forces under different conditions.

	*R. pardalis*			*S. guttatus*				
*Friction (dry)*								
	pads	belly	thigh		pads	belly	thigh	
Area at 90° (mm^2^)	65.1	72.4	108.1		20.1	38.8	119.1	
Force per area (mN mm^2^)	1.5	1.0	0.5		3.0	7.3	6.9	
Force (Area×Force per area)	98.3	74.5	53.0		60.9	284.1	820.3	
Total force (mN)		225.8				1165		
Force required (at 90°)		45.1				24.5		
*Friction (wet)*								
	pads	belly	thigh		pads	belly	thigh	
Area at 90° (mm^2^)	65.1	72.4	108.1		20.1	38.8	119.1	
Force per area (mN mm^2^)	0.2	0.3	0.3		0.1	0.1	0.3	
Force (Area×Force per area)	11.1	23.9	28.1		2.2	3.9	35.7	
Total force (mN)		63.1				41.8		
Force required (at 90°)		45.1				24.5		
*Adhesion (dry)*								
	pads	belly	thigh		pads	belly	thigh	
Area at 180° (mm^2^)	77.2	0	0		22.4	136.2	155.1	
Force per area (mN mm^2^)	1.3	1.0	0.5		2.4	1.5	0.7	
Force (Area×Force per area)	103.5	0	0		54.5	208.3	110.1	
Total force (mN)		103.5				373.0		
Force required (at 180°)		45.1				24.5		
*Adhesion (wet)*								
	pads	belly	thigh		pads	belly	thigh	
Area at 180° (mm^2^)	77.2	0	0		22.4	136.2	155.1	
Force per area (mN mm^2^)	0.2	0.7	0.3		0.1	0.3	0.3	
Force (Area×Force per area)	17.8	0	0		2.9	39.5	46.5	
Total force (mN)		17.8				88.9		
Force required (at 180°)		45.1				24.5		

Values are medians, as mean values were sometimes distorted by the occasional rogue value. All calculations were performed before reduction to a single decimal place.

### Scanning Electron Microscopy

Some torrent frogs, including members of the genus *Amolops*, have been shown to possess elongated cells, which create more direct channels between the centre of the pad and the outside [9]. *Odorrana hosii*, whose toe pad epithelium is illustrated in Figure 6C, is one such species, while *S. guttatus* only has elongated epithelial cells around the margins of its pads (Figure 6B). Recent studies on mimics of tree frog toe pads [23], [24] and insect-inspired artificial adhesive surfaces [25] have highlighted the importance of patterned surfaces for good adhesion and friction in the presence of a fluid. If drainage of excess fluid is facilitated by the channel system, repulsive hydrodynamic forces that delay close contact between pad and substrate will be minimized. However, such channels will also minimize hydrodynamic drag, which could be an important component of adhesion on flooded surfaces when there is no meniscus (air-water interface) around the edge of each toe pad to generate capillary forces.

### Effect of body mass

**Figure 8:**
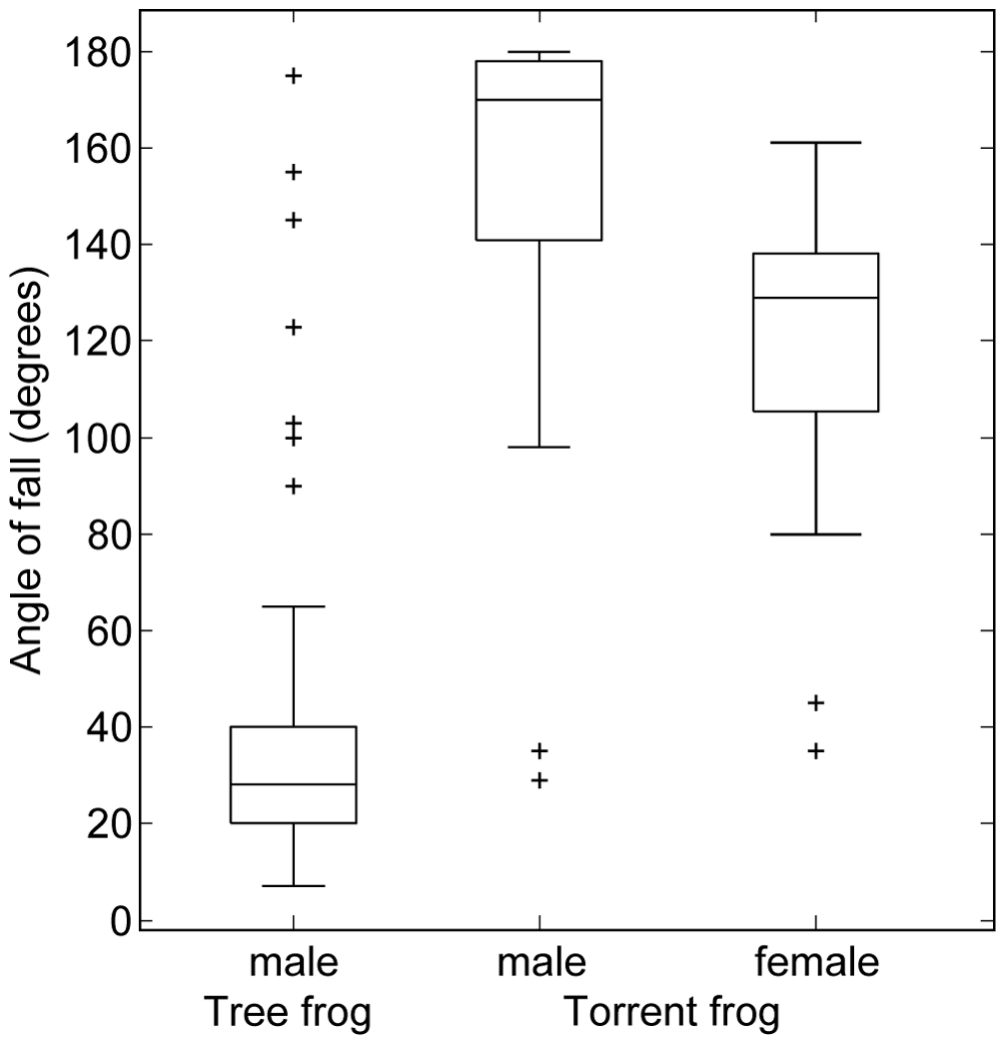
Attachment performance of male tree frogs (*R. pardalis*) and male and female torrent frogs (*S. guttatus*) on the 1125 mm rough surface at a high flow rate. Male data are from Figure 3. Angles of fall for female torrent frogs are significantly higher than those of male tree frogs (Mann-Whitney U-test, 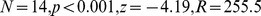), but lower than those of male torrent frogs (Mann Whitney U-test, 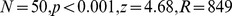).

The simplest hypothesis that might explain the differences in adhesive capabilities of tree- and torrent frogs is that it is entirely explicable in terms of body mass. We tried to find tree- and torrent frogs of equivalent size to compare, but were limited to relatively abundant species, and the species chosen were the closest match that was possible. However, the tree frogs were heavier than the torrent frogs (Table 1). It is not unreasonable to believe that this might make a difference. After all, adhesion in tree frogs scales with area [7], [26], so that larger frogs are potentially at a disadvantage. Large frog species do not normally have disproportionately larger feet [7], [13], but they often have slightly more efficient toe pads (i. e. their toe pads are capable of producing higher adhesive stresses [7], [13]). To test this hypothesis, we investigated the performance of female torrent frogs on the rotation platform. Such frogs (mean mass 8.1 g) were significantly heavier than the male tree frogs and, of course, much heavier than males of their own species. Thus, if mass were the only factor, they would be expected to perform worse than both tree frogs and male torrent frogs. Our sample size (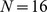) was small as these frogs were only caught occasionally. However, as Figure 8 shows, their performance on the rotating platform on the roughest surface at the highest flow rate was significantly better than that of the much lighter male tree frogs (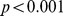, Mann Whitney U-test), although worse than that of the male torrent frogs (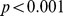, Mann-Whitney U-test). We conclude that, although body mass is a factor, it is not the main explanation for the torrent frogs' superior performance on rough wet surfaces.

### Conclusions

Our experiments clearly demonstrate that, under controlled laboratory conditions, the torrent frog, *Staurois guttatus*, adheres extremely well to rough surfaces covered in fast-flowing water, staying attached until the rotation platform is close to the upside-down position. Although the tree frog, *Rhacophorus pardalis*, also adheres well under most experimental conditions, its performance did not match the torrent frog on rough flooded surfaces. In an attempt to understand the biomechanical basis of this difference in performance, we have measured contact area, adhesive and shear forces in both frog species. These experiments illustrate the high forces that the toe pads of both species can generate, and demonstrate different strategies for staying attached to a rotating platform. Combining the data shows that, even under wet conditions where adhesive and friction forces were considerably reduced, there was more than enough force available to support the animals. Disappointingly, however, these force and contact area measurements could not be applied to the experimental situation where the torrent frogs excelled. Nevertheless, a number of important conclusions are possible that explain why torrent frogs stick so well under these extreme conditions.

1. Being small helps: Body mass was only a small factor in this particular comparison, but the much heavier torrent frog *Odorrana hosii* (mean body mass 12.0 g) did not perform well on our tilting apparatus (data not shown).
2. Use of ventral body skin as well as toe pads: Observations of the torrent frogs on the rough, wet surface indicate that they use the same strategy for adhesion as they show on the dry smooth surface. Thus, as Table 2 shows, toe pads play a relatively minor role in torrent frog adhesion at high angles of tilt. Although we failed to detect areas of thigh or belly skin that showed adaptations for adhesion (e. g. nanopillars or cells surrounded by deep channels), the large area involved means that high adhesive or shear stresses are not required. Structural adaptations, like straightened channels, which might aid rapid drainage of excess fluid from under the toe pads, characteristic of many torrent frogs [9], were poorly developed in *S. guttatus*. Given the relatively minor role of the toe pads in comparison to thigh and belly skin in our experiments, it is unlikely that they are important here.
3. High friction forces: The behavioural data demonstrated that the torrent frogs generated superior friction compared to the tree frogs on rough, wet surfaces. Indeed, their ability not to be swept off by the high flow rate showed that friction was the major factor in their superior performance.
4. Behavioural strategies: Although the few tree frogs that performed well on the rough, wet surface appeared to be using a similar strategy to the torrent frogs (i. e. using plenty of ventral body skin to aid adhesion), it did appear that not all tree frogs used optimal strategies to remain attached to a smooth surface being rotated from the horizontal to the upside-down position. While adhesion to wet, rough substrates is normal for a torrent frog, the same is not true for tree frogs. Thus, suboptimal behavioural strategies may be expected on such surfaces.

In conclusion, we do find anatomical evidence to support the idea that the toe pads of our torrent frog species have some specializations to promote better drainage of fluid from under its pads, but such adaptations seem to be restricted to the edge of the pad. Such features may lend inspiration to man-made adhesive pads which would work under similar (wet) conditions. In contrast, however, neither belly nor thigh skin of either species seems to be specialized for adhesion or friction, but as these body parts have a much greater surface area than the toe pads, they may still play an important role in adhesion and friction. We recognise, as Garland and Adolph [27] have argued, that two species comparisons are limited in their ability to allow broader conclusions about evolutionary adaptations to different habitats. However, such a two species comparison was necessary in order to test whether the torrent frog species, *Staurois guttatus* has abilities not shared by a tree frog of comparable size.

## Supporting Information

Figure S1 **Reorientation behaviour of the frogs.** A & B) Individual frames of a tree- and a torrent frog, respectively, in contact with a transparent surface at two tilt angles (45° and 135°). C & D) Plots of total contact area against rotation angle of the platform. When frogs initially faced downhill, they usually re-orientated themselves during the course of the platform rotation to face uphill again (arrows). While turning around, tree frogs often lost the contact of their belly with the surface; in contrast, torrent frogs managed to increase the contact area of their ventral surface.
(TIF)

Supplementary Materials S1 **Supplementary Tables S1 to S8. Table S1, Mann-Whitney U-tests for the comparisons of the** ***slip angles*** **for** ***R. pardalis*** **under different testing regimes.** ‘smooth’, ’30 μm’ and ‘1125 μm’ refers to the substrate roughness; ‘dry’, ‘low flow rate’ and ‘high flow rate’ refer to the amount of water on the surface. **Table S2, Mann-Whitney U-tests for the comparisons of the** ***fall angles*** **for** ***R. pardalis*** **under different testing regimes.** ‘smooth’, ’30 μm’ and ‘1125 μm’ refers to the substrate roughness; ‘dry’, ‘low flow rate’ and ‘high flow rate’ refer to the amount of water on the surface. **Table S3, Mann-Whitney U-tests for the comparisons of the** ***slip angles*** **for** ***S. guttatus*** **under different testing regimes.** ‘smooth’, ’30 μm’ and ‘1125 μm’ refers to the substrate roughness; ‘dry’, ‘low flow rate’ and ‘high flow rate’ refer to the amount of water on the surface. When the sample size was too small no z-value could be computed; when both samples contained identical values, no p-value could be obtained (‘NA’: not applicable). **Table S4, Mann-Whitney U-tests for the comparisons of the** ***slip angles*** **between the two frog species under different testing regimes.** ‘smooth’, ’30 μm’ and ‘1125 μm’ refers to the substrate roughness; ‘dry’, ‘low flow rate’ and ‘high flow rate’ refer to the amount of water on the surface.When the sample size was too small no z-value could be computed; when both samples contained identical values, no p-value could be obtained (‘NA’: not applicable). **Table S5, Mann-Whitney U-tests for the comparisons of the** ***fall angles*** **for** ***S. guttatus*** **under different testing regimes.** ‘smooth’, ’30 μm’ and ‘1125 μm’ refers to the substrate roughness; ‘dry’, ‘low flow rate’ and ‘high flow rate’ refer to the amount of water on the surface. **Table S6, Mann-Whitney U-tests for the comparisons of the** ***fall angles*** **between the two frog species under different testing regimes.** ‘smooth’, ’30 μm’ and ‘1125 μm’ refers to the substrate roughness; ‘dry’, ‘low flow rate’ and ‘high flow rate’ refer to the amount of water on the surface. **Table S7, Mann-Whitney U-tests for the comparison of** ***friction force per contact area*** **for different body parts under different conditions** ***between*** **the two frog species.** ‘smooth’, ‘0.3 μm’ and ’16 μm’ refers to the substrate roughness; ‘dry’ and ‘wet’ refer to the absence or presence of water on the surface, respectively. When the sample size was too small no z-value could be computed; when both samples contained identical values, no p-value could be obtained (‘NA’: not applicable). **Table S8, Mann-Whitney U-tests for the comparison of** ***adhesive force per contact area*** **for different body parts under different conditions between the two frog species.** ‘smooth’, ‘0.3 μm’ and ’16 μm’ refers to the substrate roughness; ‘dry’ and ‘wet’ refer to the absence or presence of water on the surface, respectively. When the sample size was too small no z-value could be computed; when both samples contained identical values, no p-value could be obtained (‘NA’: not applicable).
(PDF)

Video S1 **Torrent frog (** ***S. guttatus*** **) adhering to the rotating platform, on a rough surface (1125 μm particles) under high flow rate (ca. 4000 mL min^−1^) conditions.** Although the frog slides early on, it does not fall from the platform until the fully upside-down (180°) position is reached.
(MOV)

Video S2 **Use of the LED-illuminated platform enabled the visualisation and measurement of contact area during adhesion to a rotating glass platform.** Here, at near 180 degrees (fully upside-down position), the tree frog (*R. pardalis*) shows dynamic stability, using its toe pads alone. Under the influence of gravity, the limbs slide towards the body and are re-extended at intervals, while the fingers of the fore-limbs slide centripetally towards the hand, again being replaced in an extended position at intervals. Such ‘dancing’ behaviour allows the frog to avoid falling for it keeps the angle between pad and surface as small as possible, avoiding detachment by peeling (see [17] for a full explanation).
(MPEG)

Video S3 **Comparison of typical turning behaviour of the tree frog (** ***R. pardalis*** **) (upper video and animated graph) and the torrent frog (** ***S. guttatus*** **) (lower video and animated graph) on the LED-illuminated platform.** Such turning takes place during rotation whenever the frogs find themselves facing downhill. Typically, body turning in the tree frog results in loss of body contact, so that the frog is subsequently attaching by its toe pads alone. However, torrent frogs usually turn without permanently losing body contact. Indeed, body contact area increases with the angle of tilt. The graphs show total contact area against platform rotation angle (a measure of time), the moving vertical line indicating the position in the video.
(MPEG)
