# Supplementary figures and images for: Sticking under Wet Conditions: The Remarkable Attachment Abilities of the Torrent Frog, Staurois guttatus
Source: PLoS One. 2013 Sep 25;8(9):e73810. doi: 10.1371/journal.pone.0073810 (PMC3783468; doi:10.1371/journal.pone.0073810)

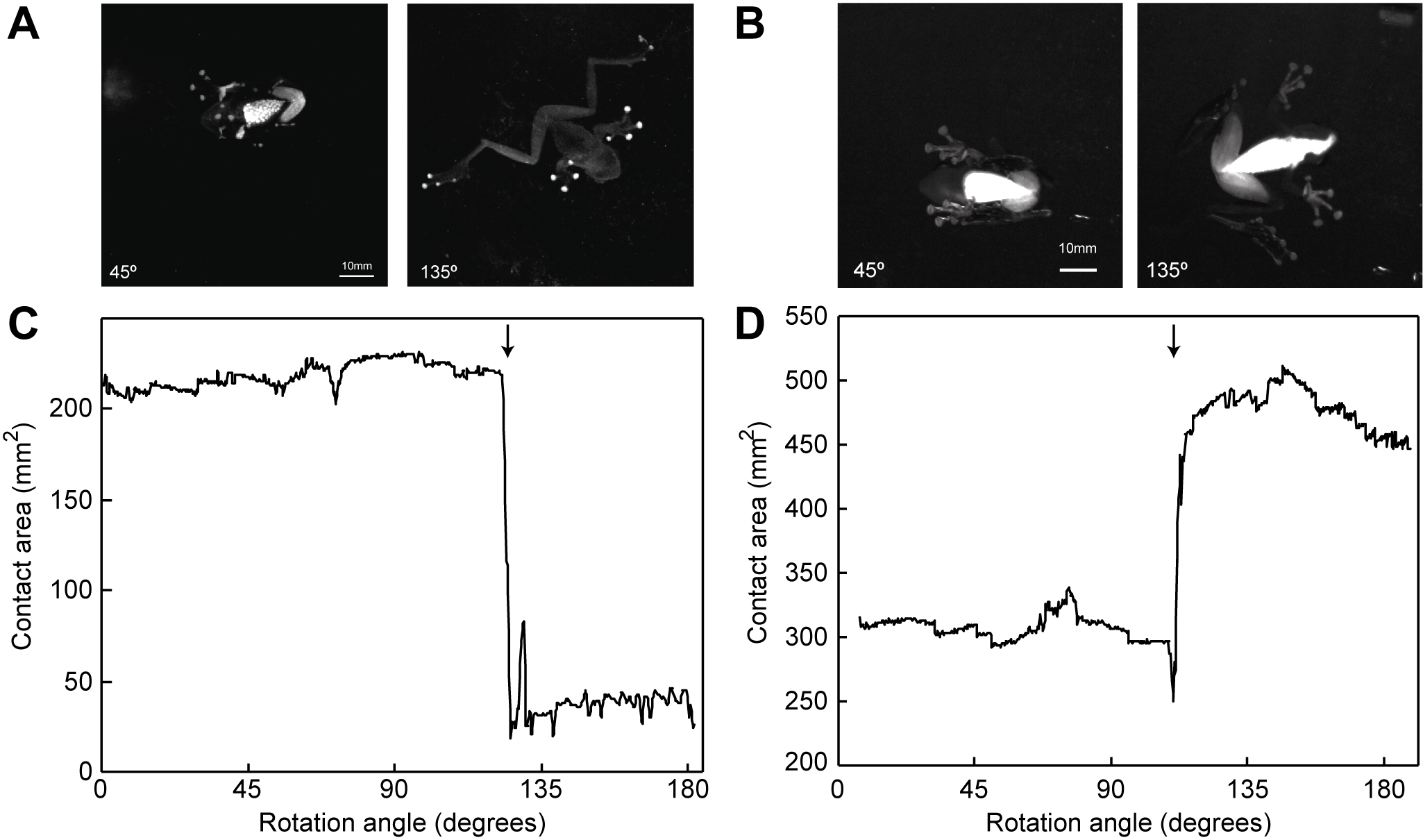

Supplement: Figure S1 — Reorientation behaviour of the frogs. A & B) Individual frames of a tree- and a torrent frog, respectively, in contact with a transparent surface at two tilt angles (45° and 135°). C & D) Plots of total contact area against rotation angle of the platform. When frogs initially faced downhill, they usually re-orientated themselves during the course of the platform rotation to face uphill again (arrows). While turning around, tree frogs often lost the contact of their belly with the surface; in contrast, torrent frogs managed to increase the contact area of their ventral surface. (TIF) [file pone.0073810.s001.tif]
