# Supplementary material for: Sticking under Wet Conditions: The Remarkable Attachment Abilities of the Torrent Frog, Staurois guttatus
Source: PLoS One. 2013 Sep 25;8(9):e73810. doi: 10.1371/journal.pone.0073810 (PMC3783468; doi:10.1371/journal.pone.0073810)
Supplement: Supplementary Materials S1 — Supplementary Tables S1 to S8. Table S1, Mann-Whitney U-tests for the comparisons of the slip angles for R. pardalis under different testing regimes. ‘smooth’, ’30 μm’ and ‘1125 μm’ refers to the substrate roughness; ‘dry’, ‘low flow rate’ and ‘high flow rate’ refer to the amount of water on the surface. Table S2, Mann-Whitney U-tests for the comparisons of the fall angles for R. pardalis under different testing regimes. ‘smooth’, ’30 μm’ and ‘1125 μm’ refers to the substrate roughness; ‘dry’, ‘low flow rate’ and ‘high flow rate’ refer to the amount of water on the surface. Table S3, Mann-Whitney U-tests for the comparisons of the slip angles for S. guttatus under different testing regimes. ‘smooth’, ’30 μm’ and ‘1125 μm’ refers to the substrate roughness; ‘dry’, ‘low flow rate’ and ‘high flow rate’ refer to the amount of water on the surface. When the sample size was too small no z-value could be computed; when both samples contained identical values, no p-value could be obtained (‘NA’: not applicable). Table S4, Mann-Whitney U-tests for the comparisons of the slip angles between the two frog species under different testing regimes. ‘smooth’, ’30 μm’ and ‘1125 μm’ refers to the substrate roughness; ‘dry’, ‘low flow rate’ and ‘high flow rate’ refer to the amount of water on the surface.When the sample size was too small no z-value could be computed; when both samples contained identical values, no p-value could be obtained (‘NA’: not applicable). Table S5, Mann-Whitney U-tests for the comparisons of the fall angles for S. guttatus under different testing regimes. ‘smooth’, ’30 μm’ and ‘1125 μm’ refers to the substrate roughness; ‘dry’, ‘low flow rate’ and ‘high flow rate’ refer to the amount of water on the surface. Table S6, Mann-Whitney U-tests for the comparisons of the fall angles between the two frog species under different testing regimes. ‘smooth’, ’30 μm’ and ‘1125 μm’ refers to the substrate roughness; ‘dry’, ‘low flow rate’ and ‘high flow rate’ refer to th [file pone.0073810.s002.pdf]

**Table S 1.** Mann-Whitney U-tests for the comparisons of the *slip angles* for *R. pardalis* under different testing regimes. 'smooth', '30 µm' and '1125 µm' refers to the substrate roughness; 'dry', 'low flow rate' and 'high flow rate' refer to the amount of water on the surface.

| No. | Substrate condition | Test | $z$ | $R$ | $p$ |
|---|---|---|---|---|---|
| 1 | smooth | dry *vs.* low flow rate | 1.39 | 2602 | >0.05 |
| 2 | smooth | dry *vs.* high flow rate | 9.18 | 3775 | <0.001 |
| 3 | smooth | low *vs.* high flow rate | 9.07 | 3775 | <0.001 |
| 4 | 30 µm | dry *vs.* low flow rate | NA | 2525 | NA |
| 5 | 30 µm | dry *vs.* high flow rate | 9.23 | 3775 | <0.001 |
| 6 | 30 µm | low *vs.* high flow rate | 9.23 | 3775 | <0.001 |
| 7 | 1125 µm | dry *vs.* low flow rate | -2.17 | 2377 | =0.09 |
| 8 | 1125 µm | dry *vs.* high flow rate | 8.96 | 3772 | <0.001 |
| 9 | 1125 µm | low *vs.* high flow rate | 9.17 | 3775 | <0.001 |
| 10 | dry | smooth *vs.* 30 µm | -0.98 | 2500 | >0.05 |
| 11 | dry | smooth *vs.* 1125 µm | 2.2 | 2676 | =0.08 |
| 12 | dry | 30 µm *vs.* 1125 µm | 2.72 | 2700 | <0.05 |
| 13 | low flow rate | smooth *vs.* 30 µm | -2.02 | 2425 | >0.05 |
| 14 | low flow rate | smooth *vs.* 1125 µm | -1.34 | 2451 | >0.05 |
| 15 | low flow rate | 30 µm *vs.* 1125 µm | 0.98 | 2550 | >0.05 |
| 16 | high flow rate | smooth *vs.* 30 µm | -6.28 | 1618 | <0.001 |
| 17 | high flow rate | smooth *vs.* 1125 µm | -5.88 | 1674 | <0.001 |
| 18 | high flow rate | 30 µm *vs.* 1125 µm | -1.95 | 2244 | >0.05 |

**Table S 2.** Mann-Whitney U-tests for the comparisons of the *fall angles* for *R. pardalis* under different testing regimes. 'smooth', '30 µm' and '1125 µm' refers to the substrate roughness; 'dry', 'low flow rate' and 'high flow rate' refer to the amount of water on the surface.

| No. | Substrate condition | Test | $z$ | $R$ | $p$ |
|---|---|---|---|---|---|
| 1 | smooth | dry *vs.* low flow rate | 3.28 | 2994 | <0.01 |
| 2 | smooth | dry *vs.* high flow rate | 6.33 | 3439 | <0.001 |
| 3 | smooth | low *vs.* high flow rate | 4.63 | 3196 | <0.001 |
| 4 | 30 µm | dry *vs.* low flow rate | -4.88 | 1860 | <0.001 |
| 5 | 30 µm | dry *vs.* high flow rate | 8.64 | 3775 | <0.001 |
| 6 | 30 µm | low *vs.* high flow rate | 8.84 | 3775 | <0.001 |
| 7 | 1125 µm | dry *vs.* low flow rate | -7.35 | 1464 | <0.001 |
| 8 | 1125 µm | dry *vs.* high flow rate | 6.91 | 3527 | <0.001 |
| 9 | 1125 µm | low *vs.* high flow rate | 7.94 | 3674 | <0.001 |
| 10 | dry | smooth *vs.* 30 µm | 3.08 | 2964 | <0.01 |
| 11 | dry | smooth *vs.* 1125 µm | 8.36 | 3732 | <0.001 |
| 12 | dry | 30 µm *vs.* 1125 µm | 6.32 | 3440 | <0.001 |
| 13 | low flow rate | smooth *vs.* 30 µm | -5.19 | 1808 | <0.001 |
| 14 | low flow rate | smooth *vs.* 1125 µm | -2.23 | 2205 | =0.07 |
| 15 | low flow rate | 30 µm *vs.* 1125 µm | 3.24 | 2955 | <0.01 |
| 16 | high flow rate | smooth *vs.* 30 µm | 3.03 | 2964 | <0.01 |
| 17 | high flow rate | smooth *vs.* 1125 µm | 2.12 | 2833 | >0.05 |
| 18 | high flow rate | 30 µm *vs.* 1125 µm | -2.41 | 2176 | <0.05 |

**Table S 3.** Mann-Whitney U-tests for the comparisons of the *slip angles* for *S. guttatus* under different testing regimes. 'smooth', '30 µm' and '1125 µm' refers to the substrate roughness; 'dry', 'low flow rate' and 'high flow rate' refer to the amount of water on the surface. When the sample size was too small no z-value could be computed; when both samples contained identical values, no p-value could be obtained ('NA': not applicable).

| No. | Substrate condition | Test | $z$ | $R$ | $p$ |
|---|---|---|---|---|---|
| 1 | smooth | dry *vs.* low flow rate | 1.74 | 2600 | >0.05 |
| 2 | smooth | dry *vs.* high flow rate | -9.11 | 1081 | <0.001 |
| 3 | smooth | low *vs.* high flow rate | -8.98 | 1081 | <0.001 |
| 4 | 30 µm | dry *vs.* low flow rate | NA | 2525 | NA |
| 5 | 30 µm | dry *vs.* high flow rate | 8.92 | 3725 | <0.001 |
| 6 | 30 µm | low *vs.* high flow rate | 8.92 | 3725 | <0.001 |
| 7 | 1125 µm | dry *vs.* low flow rate | -1.41 | 2475 | >0.05 |
| 8 | 1125 µm | dry *vs.* high flow rate | -7.71 | 1343 | <0.001 |
| 9 | 1125 µm | low *vs.* high flow rate | -7.93 | 1328 | <0.001 |
| 10 | dry | smooth *vs.* 30 µm | NA | 2525 | NA |
| 11 | dry | smooth *vs.* 1125 µm | 1.41 | 2575 | >0.05 |
| 12 | dry | 30 µm *vs.* 1125 µm | 1.41 | 2575 | >0.05 |
| 13 | low flow rate | smooth *vs.* 30 µm | -1.74 | 2450 | >0.05 |
| 14 | low flow rate | smooth *vs.* 1125 µm | -1.74 | 2450 | >0.05 |
| 15 | low flow rate | 30 µm *vs.* 1125 µm | NA | 2525 | NA |
| 16 | high flow rate | smooth *vs.* 30 µm | -5.06 | 1542 | <0.001 |
| 17 | high flow rate | smooth *vs.* 1125 µm | -7.53 | 1183 | <0.001 |
| 18 | high flow rate | 30 µm *vs.* 1125 µm | 3.24 | 2752 | <0.01 |

**Table S 4.** Mann-Whitney U-tests for the comparisons of the *slip angles* between the two frog species under different testing regimes. 'smooth', '30 µm' and '1125 µm' refers to the substrate roughness; 'dry', 'low flow rate' and 'high flow rate' refer to the amount of water on the surface.When the sample size was too small no z-value could be computed; when both samples contained identical values, no p-value could be obtained ('NA': not applicable).

| No. | Substrate condition | Test | $z$ | $R$ | $p$ |
|---|---|---|---|---|---|
| 1 | smooth | dry | -0.98 | 2500 | >0.05 |
| 2 | smooth | low flow rate | -0.31 | 2505 | >0.05 |
| 3 | smooth | high flow rate | 3.99 | 2773 | <0.001 |
| 4 | 30 µm | dry | NA | 2525 | NA |
| 5 | 30 µm | low flow rate | NA | 2525 | NA |
| 6 | 30 µm | high flow rate | -3.52 | 2017 | <0.01 |
| 7 | 1125 µm | dry | -1.67 | 2404 | >0.05 |
| 8 | 1125 µm | low flow rate | -0.98 | 2500 | >0.05 |
| 9 | 1125 µm | high flow rate | 4.95 | 2988 | <0.001 |

**Table S 5.** Mann-Whitney U-tests for the comparisons of the *fall angles* for *S. guttatus* under different testing regimes. 'smooth', '30 µm' and '1125 µm' refers to the substrate roughness; 'dry', 'low flow rate' and 'high flow rate' refer to the amount of water on the surface.

| No. | Substrate condition | Test | $z$ | $R$ | $p$ |
|---|---|---|---|---|---|
| 1 | smooth | dry *vs.* low flow rate | -1.85 | 2308 | >0.05 |
| 2 | smooth | dry *vs.* high flow rate | -5.99 | 1428 | <0.001 |
| 3 | smooth | low *vs.* high flow rate | -7.53 | 1239 | <0.001 |
| 4 | 30 µm | dry *vs.* low flow rate | -6.84 | 1596 | <0.001 |
| 5 | 30 µm | dry *vs.* high flow rate | 3.61 | 3049 | <0.001 |
| 6 | 30 µm | low *vs.* high flow rate | 8.3 | 3685 | <0.001 |
| 7 | 1125 µm | dry *vs.* low flow rate | -7.7 | 1435 | <0.001 |
| 8 | 1125 µm | dry *vs.* high flow rate | 6.13 | 3152 | <0.001 |
| 9 | 1125 µm | low *vs.* high flow rate | -4.34 | 1727 | <0.001 |
| 10 | dry | smooth *vs.* 30 µm | 5.44 | 3289 | <0.001 |
| 11 | dry | smooth *vs.* 1125 µm | 7.7 | 3624 | <0.001 |
| 12 | dry | 30 µm *vs.* 1125 µm | 3.28 | 3001 | <0.01 |
| 13 | low flow rate | smooth *vs.* 30 µm | -0.45 | 2479 | >0.05 |
| 14 | low flow rate | smooth *vs.* 1125 µm | 1.03 | 2641 | >0.05 |
| 15 | low flow rate | 30 µm *vs.* 1125 µm | 1.45 | 2684 | >0.05 |
| 16 | high flow rate | smooth *vs.* 30 µm | 1.34 | 2414 | >0.05 |
| 17 | high flow rate | smooth *vs.* 1125 µm | -4.17 | 1619 | <0.001 |
| 18 | high flow rate | 30 µm *vs.* 1125 µm | 5.84 | 3112 | <0.001 |

**Table S 6.** Mann-Whitney U-tests for the comparisons of the *fall angles* between the two frog species under different testing regimes. 'smooth', '30 µm' and '1125 µm' refers to the substrate roughness; 'dry', 'low flow rate' and 'high flow rate' refer to the amount of water on the surface.

| No. | Substrate condition | Test | $z$ | $R$ | $p$ |
|---|---|---|---|---|---|
| 1 | smooth | dry | -1.97 | 2260 | >0.05 |
| 2 | smooth | low flow rate | -6.07 | 1694 | <0.001 |
| 3 | smooth | high flow rate | 2.33 | 2549 | =0.059 |
| 4 | 30 µm | dry | 1.93 | 2804 | >0.05 |
| 5 | 30 µm | low flow rate | -1.3 | 2385 | >0.05 |
| 6 | 30 µm | high flow rate | -6.36 | 1605 | <0.001 |
| 7 | 1125 µm | dry | -1.8 | 2264 | >0.05 |
| 8 | 1125 µm | low flow rate | -3.23 | 2095 | <0.01 |
| 9 | 1125 µm | high flow rate | 7.55 | 3349 | <0.001 |

**Table S 7.** Mann-Whitney U-tests for the comparison of *friction force per contact area* for different body parts under different conditions *between* the two frog species. 'smooth', '0.3 µm' and '16 µm' refers to the substrate roughness; 'dry' and 'wet' refer to the absence or presence of water on the surface, respectively. When the sample size was too small no z-value could be computed; when both samples contained identical values, no p-value could be obtained ('NA': not applicable).

| No. | Body part | Substrate condition | $N$ | $z$ | $R$ | $p$ |
|---|---|---|---|---|---|---|
| 1 | pad | dry, smooth | 34 | -2.28 | 1129 | <0.05 |
| 2 | pad | low flow rate, smooth | 11 | 0.37 | 168 | >0.05 |
| 3 | belly | dry, smooth | 10 | 3.87 | 45 | <0.001 |
| 4 | belly | low flow rate, smooth | 13 | 1.81 | 182 | =0.06 |
| 5 | thigh | dry, smooth | 11 | 1.93 | 116 | =0.053 |
| 6 | thigh | low flow rate, smooth | 12 | 0.25 | 155 | >0.05 |
| 7 | pad | dry, 0.3 µm | 6 | NA | 54 | >0.05 |
| 8 | pad | low flow rate, 0.3 µm | 4 | NA | 41 | <0.01 |
| 9 | pad | dry, 16 µm | 8 | NA | 83 | >0.05 |
| 10 | pad | low flow rate, 16 µm | 8 | NA | 95 | <0.01 |

**Table S 8.** Mann-Whitney U-tests for the comparison of *adhesive force per contact area* for different body parts under different conditions between the two frog species. 'smooth', '0.3 µm' and '16 µm' refers to the substrate roughness; 'dry' and 'wet' refer to the absence or presence of water on the surface, respectively. When the sample size was too small no z-value could be computed; when both samples contained identical values, no p-value could be obtained ('NA': not applicable).

| No. | Body part | Substrate condition | $N$ | $z$ | $R$ | $p$ |
|---|---|---|---|---|---|---|
| 1 | pad | dry, smooth | 34 | -2.2 | 1129 | <0.05 |
| 2 | pad | low flow rate, smooth | 11 | 0.37 | 168 | >0.05 |
| 3 | belly | dry, smooth | 10 | 0.79 | 162 | >0.05 |
| 4 | belly | low flow rate, smooth | 13 | -1.53 | 193 | >0.05 |
| 5 | thigh | dry, smooth | 11 | 0.39 | 186 | >0.05 |
| 6 | thigh | low flow rate, smooth | 12 | 0.019 | 199 | >0.05 |
| 7 | pad | dry, 0.3 µm | 6 | NA | 54 | =0.054 |
| 8 | pad | low flow rate, 0.3 µm | 4 | NA | 41 | <0.05 |
| 9 | pad | dry, 16 µm | 8 | NA | 83 | =0.08 |
| 10 | pad | low flow rate, 16 µm | 8 | NA | 95 | =0.09 |
